# Dose-Effect Relationship of Chitosan and Danshen Combined Injection for Fallopian Tube Recanalization

**DOI:** 10.3389/fphar.2022.935117

**Published:** 2022-06-14

**Authors:** Chen Huang, Qiong Wu, Jiabin Liang, Qian Wang, Xueping He, Yanqiu Xie, Yanbing Lu, Jianfen Su, Yutuan Tang

**Affiliations:** ^1^ Department of Minimally Invasive Interventional Radiology, Guangzhou Panyu Central Hospital, Guangzhou, China; ^2^ Medical Imaging Institute of Panyu, Guangzhou, China; ^3^ Department of Medical Aesthetic, The First Hospital of Xi’an, Xi’an, China; ^4^ Provincial Key Laboratory of Biotechnology of Shaanxi, Key Laboratory of Resource Biology and Modern Biotechnology in Western China, Faculty of Life Science, Northwest University, Xi’an, China; ^5^ Guangzhou University of Chinese Medicine, Guangzhou University Town, Guangzhou, China

**Keywords:** chitosan, danshen, dose-effect relationships, drug delivery, fallopian tube recanalization

## Abstract

**Objectives:** This study examined the dose-effect relationship of chitosan and danshen combined injections on the long-term prevention of fallopian tube re-obstructions, with increased pregnancy rates in infertile women.

**Methods:** High-performance liquid chromatography was used to determine the content changes of combined chitosan and danshen injection. Two hundred and eighty patients with fallopian tube obstructions were randomly assigned to four groups. Group A (control group, saline), Group B (2 ml chitosan, 4 ml danshen), Group C (2 ml chitosan, 10 ml danshen), and Group D (1 ml chitosan, 10 ml danshen). Injections were administered after tubal recanalization.

**Results:** The effective constituent of chitosan and danshen injection was stable. Tubal patency rate was 94.2% and 87.3% in Group C after 1 and 3 years, respectively, which was significantly higher than Groups A (38.6%, 31.5%), B (73.5%, 64.1%), and D (68.5%, 50.7%). Intrauterine pregnancy rates were 61.8% and 79.4% in Group C after 1 and 3 years, respectively, and were significantly higher than Groups A (31.8%, 34.8%), B (40.1%, 62.5%), and D (38.5%, 58.5%) (*p* < 0.05).

**Conclusion:** Combined Chitosan and danshen injections prevented tubal obstruction and increased pregnancy rates for long periods using an optimal ratio of 1 part chitosan and 5 parts danshen.

## 1 Introduction

Chitosan, a polysaccharide acquired from the shells of marine creatures, is a non-toxic, biocompatible, and biodegradable material with mucoadhesion and permeation enhancements (Gao et al., 2021). Carboxymethyl chitin is the primary active ingredient. Chitosan has desirable antimicrobial properties, promotes wound healing and is used in various clinical applications. Research (Kou et al., 2022) has shown that the “molecular chain configuration” model should be used to explain the possible interactive mechanisms among the different molecular weight chitosan molecules. With these beneficial bioactivities in mind, chitosan can be used in surgery and tissue engineering and is becoming an important medical polymer.

Danshen comes from the dried roots and rhizomes of *Salvia miltiorrhiza* Bge and is a traditional Chinese medicinal. The chemical constituents of Danshen include salvianolic acid A and protocatechualdehyde, as well as several other chemicals. Our previous research (Huang et al., 2019) has shown that combined chitosan and danshen injections could decrease tubal re-occlusion post-injection in infertile women with fallopian tube occlusions, increasing pregnancy rates for brief periods.

Infertility occurs in approximately 15% of reproductive-aged couples worldwide (Hanson et al., 2017). Fallopian tube obstruction can lead to reduced fertility among women, accounting for 25–∼ 45% of all causes of infertility (Fields et al., 2013; Practice Committee of the American Society for Reproductive M, 2015). Although microsurgery is becoming more common (leading to a reduction in adhesions caused by intraabdominal cavity laparotomy), adhesions cannot be completely prevented through the microsurgical technique (Xiao et al., 2014).

Interventional fallopian tube recanalization (FTR) is extremely effective for treating tubal obstruction in cases of infertility with a technical success rate as high as 90% (Ikechebelu et al., 2018; Gao et al., 2019). One-year pregnancy rate following FTR is approximately 41% (Kohi, 2021). FTR is an alternative to vitro fertilization (IVF) and intracytoplasmic sperm injection (ICSI) and should be top-of-mind in the setting of infertility due to fallopian tube obstruction (Kohi, 2021). FTR is a safe treatment option in patients with fallopian tube obstruction, with high technical success rate and increased chances of pregnancy. It is prior to more invasive and expensive treatments (Anil et al., 2011).

FTR had become the universally accepted therapeutic option of fallopian tube obstruction in the last 2 decades (Verma et al., 2009; Anil et al., 2011). However, more than 20% of patients have poor outcomes following interventional recanalization (Shen et al., 2020). Niuniu Sun showed that fallopian tube recanalization by ozone perfusion could effectively increase the postoperative pregnancy rate, but there was no significant difference in the recanalization success rate by ozone perfusion (Sun et al., 2017).

In previous clinical studies, we have showed that chitosan and danshen combined injection maybe reduce the rate of tubal postoperative re-occlusion preliminarily (Huang et al., 2019).In this study, we examined the curative rates and dose effects of combined injections in the prevention of fallopian tube re-obstruction after recanalization.

## 2 Materials and Methods

### 2.1 Synergistic Research on Combined Chitosan and Danshen Injection

Chitosan (12080311, China Pharmaceutical and Biological Products Inspection Institute, China) and danshen (110855-200809, China Pharmaceutical and Biological Products Inspection Institute, China) were combined. The available contents of these natural remedies were evaluated using high-performance liquid chromatography (DGU-20a5, Shimadzu, Japan) immediately and after 10 min.

Chromatographic conditions used were as follows: Inertsil C18 column = 4.6 × 250 mm, 5 μm; flow rate = 1.0 ml/min; dual wavelength detection = 210–280 nm; sample quantity = 10 μl; mobile phase elutions included (A) 1% phosphoric acid, and (B) methanol gradient elution.

Chitosan (1 ml) and danshen injections (5 ml) were combined with ultrapure water at 1 and 5 ml and diluted 2 times into the liquid phase at 0, 10, 30 min. The main ingredients were sodium danshen, protocatechuic aldehyde, and carboxymethyl chitin and recorded as peak times and peak areas on the chromatograph chart. Each condition was assessed at least in triplicate.

### 2.2 Combined Chitosan and Danshen Injections in Fallopian Tube Recanalization

This prospective study was conducted at our hospital between 2014 and 2018. The study was approved by the medical ethics committee of Guangzhou Panyu Central Hospital (Reference number: P20140042). Informed consent was obtained from all patients prior to participation in the study.

The Inclusion criteria were 1) females of childbearing age (between 22 and 45 years), and 2) a Fallopian tube isthmus or interstitial obstruction diagnosed without adhesions of the fimbriated tubal extremity, determined on laparoscopic examination or salpingography. The exclusion criteria were 1) pelvic infection, congenital physiological defects or malformations, infertility caused by genetic factors, endocrine factors [follicle-stimulating hormone (FSH) or luteinizing hormone [LH] or other hormones] or immune factors, 2) endometriosis, uterine myoma, uterine hypoplasia, tubal ligation, or pelvic tuberculosis, 3) male partners were diagnosed with reproductive dysfunction (Huang et al., 2019; Shen et al., 2020).

A total of 280 eligible patients were enrolled between 2014 and 2018. Patients were randomized into four groups of 70 patients each, including Group A group (control group, saline only), Group B (2 ml chitosan and 4 ml danshen [1:2]), Group C (2 ml chitosan and 10 ml danshen [1:5]), and Group D group (1 ml chitosan and 10 ml danshen [1:10]). The groups were blinded to the nature of the anti-adhesion agents they had received. All groups underwent coaxial catheterizations either alone (Groups A) or after followed chitosan and danshen combined injections (Groups B, C, and D).

First, specific reproductive hormone levels (FSH, LH, estrogen, and progesterone) were measured and assessed. Interventional recanalizations were performed within 3–7 days after the end of menstruation. The procedures were performed under digital subtraction angiography (DSA) with an OEC 9600 C-arm X-ray machine (General Electric, United States). A 20 ml dose of the contrast agent (Iopromide, Ultravist, Bayer, Germany) was injected into the uterine cavity to identify the uterine shape and position of the cornu uteri, as well as to confirm the obstructed portion(s) of the fallopian tube. Then, the guidewire and catheter were directed to the cornu uteri on the affected side. Finally, a saline solution (10 ml) was administered to patients in Group A. For Groups B, C, and D, a 2 ml chitosan, 4 ml danshen solution, a 2 ml chitosan, 10 ml danshen solution, and a 1 ml chitosan and 10 ml danshen solution were administered, respectively.

Through preoperative salpingography, 400 obstructed fallopian tubes were discovered.All the patients were followed for 1 and 3 years to assess tubal patency maintenance using salpingography. Ultrasound was used to ascertain pregnancy, which was also determined to be intrauterine pregnancy rather than ectopic pregnancy during the 1- and 3 year follow-up times. Patients who did not return for follow-up examinations were contacted by telephone.

#### 2.2.1 Salpingography Definitions

i. Unobstructed: Huang et al. (2019) both sides of the fallopian tubes are clearly shown during the procedure and visible contrast agent can be seen diffusing through the pelvic cavity.
ii. Completely obstructed: Partial or no development of the middle and distal end of the fallopian tube, and no contrast agent can be seen diffusing through the pelvic cavity.
iii. Partially obstructed: The contrast agent is seen passing through the fallopian tube with partial flow into the pelvic cavity in a heterogeneous manner. Additionally, fallopian tubes often form irregular configurations.

### 3 Statistical Analyses

The data from each group were collected and analyzed using SPSS 11.5 software (IBM SPSS China, Shanghai, China). The enumeration data were checked and corrected using the Chi-square test (x^2^), whereas the measurement data were calculated as the mean (X) ± standard deviation. The one-tailed *t*-test was also used, and significant differences were only identified if the *p*-value was < 0.05.

## 4 Results

### 4.1 The Relative Effective Constituents of Chitosan and Danshen Injections Were Stable

**FIGURE 1:**
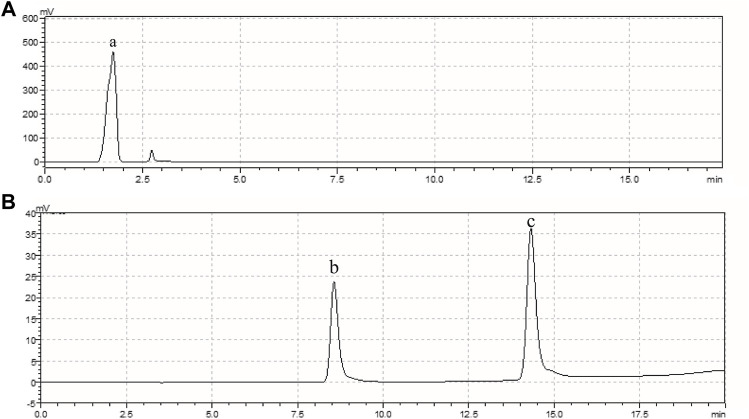
Chromatogram Chart. **(A)**. The 210 nm wavelength shows the peak for carboxymethyl chitin **(a)**. **(B)**. The 280 nm wavelength shows peaks for salvianic acid A sodium **(b)** and protocatechualdehyde **(c)**.

Chromatographic readings were detected as shown in Figure 1; Table 1. Peak area ratios between main danshen and chitosan ingredients were shown in Table 2. Table 3 showed the results after statistical analysis (*t*-tests); no statistical significance was found between the groups. The chitosan and danshen mixture before and after 0 and 10 min had no significant differences between the content of each ingredient, as shown in Table 3.

**TABLE 1:** Precision table.

	Danshen		Chitosan	Combined chitosan and danshen		
	Sodium danshen	Protocatechuic aldehyde	Carboxymethyl chitin	Sodium danshen	Protocatechuic aldehyde	Carboxymethyl chitin
RSD/%	0.8	1.4	1.2	0.9	1.8	1.1

RSD, relative standard deviation.

**TABLE 2:** Peak area ratios between the main ingredients of danshen and medicinal chitosan injections alone and combined.

		Danshen (%)		Chitosan (%)		Combined chitosan and danshen (%)	
Time/min	N	Sodium danshen	Protocatechuic aldehyde	Carboxymethyl chitin	Sodium danshen	Protocatechuic aldehyde	Carboxymethyl chitin
0	1	100.0	100.0	100.0	101.0	99.4	101.9
	2	102.1	101.7	100.8	101.6	100.2	99.5
	3	101.4	99.7	99.7	100.8	99.7	100.4
Mean		101.2	100.5	100.2	101.1	99.8	100.6
SD		1.1	1.1	0.6	0.4	0.4	1.2
10	1	100.3	100.9	99.5	100.5	100.2	100.1
	2	100.8	99.9	99.7	101.7	100.8	99.3
	3	101.5	98.9	100.1	100.3	101.3	101.3
Mean		100.9	99.9	99.8	100.8	100.8	100.2
SD		0.6	1.0	0.3	0.8	0.6	1.0

N, number; SD, standard deviation.

**TABLE 3:** Statistical analysis of the contents of the main ingredients (*t*-test).

	Before mix (P)	After mix (P)	0 min (P)	10 min (P)
Sodium Danshen	0.35	0.22	0.19	0.55
Protocatechuic aldehyde	0.72	0.69	0.10	0.48
Carboxymethyl chitin	0.26	0.75	0.29	0.18

### 4.2 Combined Chitosan and Danshen Injections Prevent Tubal Re-Obstructions and Increase Pregnancy Rates

Two hundred and sixty-three of them completed 3 years of follow-up. There were no statistically significant differences in the mean ages of the study groups (*p* > 0.05). The differences in the hormone levels (estrogen and progesterone, FSH, and LH) were not statistically different before surgery (*p* > 0.05). No statistical differences were also found among the groups regarding the location of the fallopian tube obstruction or the positions of those obstructions (one or both sides) preoperatively (*p* > 0.05). Baseline patient characteristics are listed in Table 4.

**TABLE 4:** Patient characteristics.

Characteristics	Group A (*n* = 66)	Group B (n = 64)	Group C (*n* = 68)	Group D (*n* = 65)	*p*
Age, mean ± SD (y)	30.05 ± 5.18	30.08 ± 4.64	29.97 ± 4.37	30.03 ± 5.32	>0.05
estrogen (ng/L)	178.49 ± 39.59	178.51 ± 37.77	178.49 ± 29.90	178.50 ± 34.76	>0.05
progesterone (μg/L)	14.43 ± 6.42	14.41 ± 6.43	14.42 ± 6.06	14.44 ± 5.26	>0.05
follicle stimulating hormone (U/l)	9.00 ± 3.56	9.01 ± 3.87	9.02 ± 3.81	9.02 ± 3.04	>0.05
luteinizing hormone (U/l)	29.06 ± 14.86	29.07 ± 16.58	29.09 ± 16.70	29.04 ± 17.48	>0.05
Patients of obstruction in both fallopian tubes (n)	36	33	34	34	>0.05
Patients of obstruction in Isthmus of fallopian tube(n)	15	14	16	17	>0.05

**FIGURE 2:**
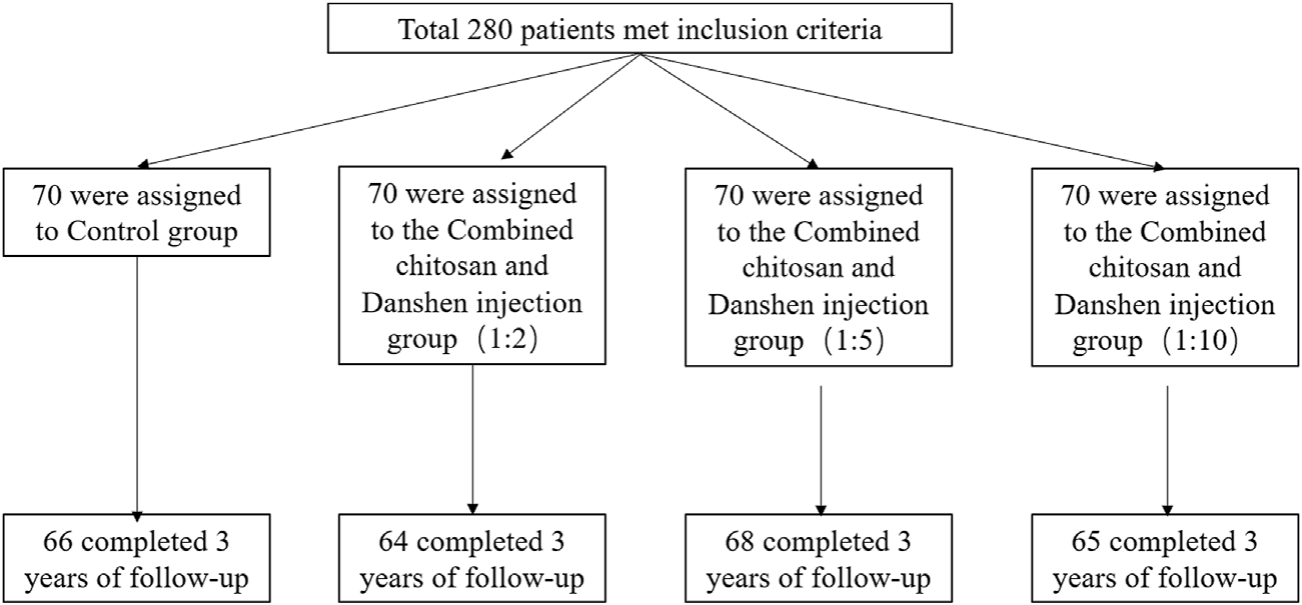
Flow chart illustrating patient recruitment and analysis.

Seventeen of the enrolled patients were lost to follow-up, including 4 in Group A group, six in Group B, 2 in Group C, and 5 in group D (Figure 2). No statistically significant differences were found between the patients who had completed the study and those who were lost to follow-up with respect to age, hormone level, obstruction location, and obstruction position preoperatively (one or both sides of the fallopian tube).

#### 4.2.1 Tubal Patency Rates in Non pregnant Patients at 1- and 3 years Post-Injection

**FIGURE 3:**
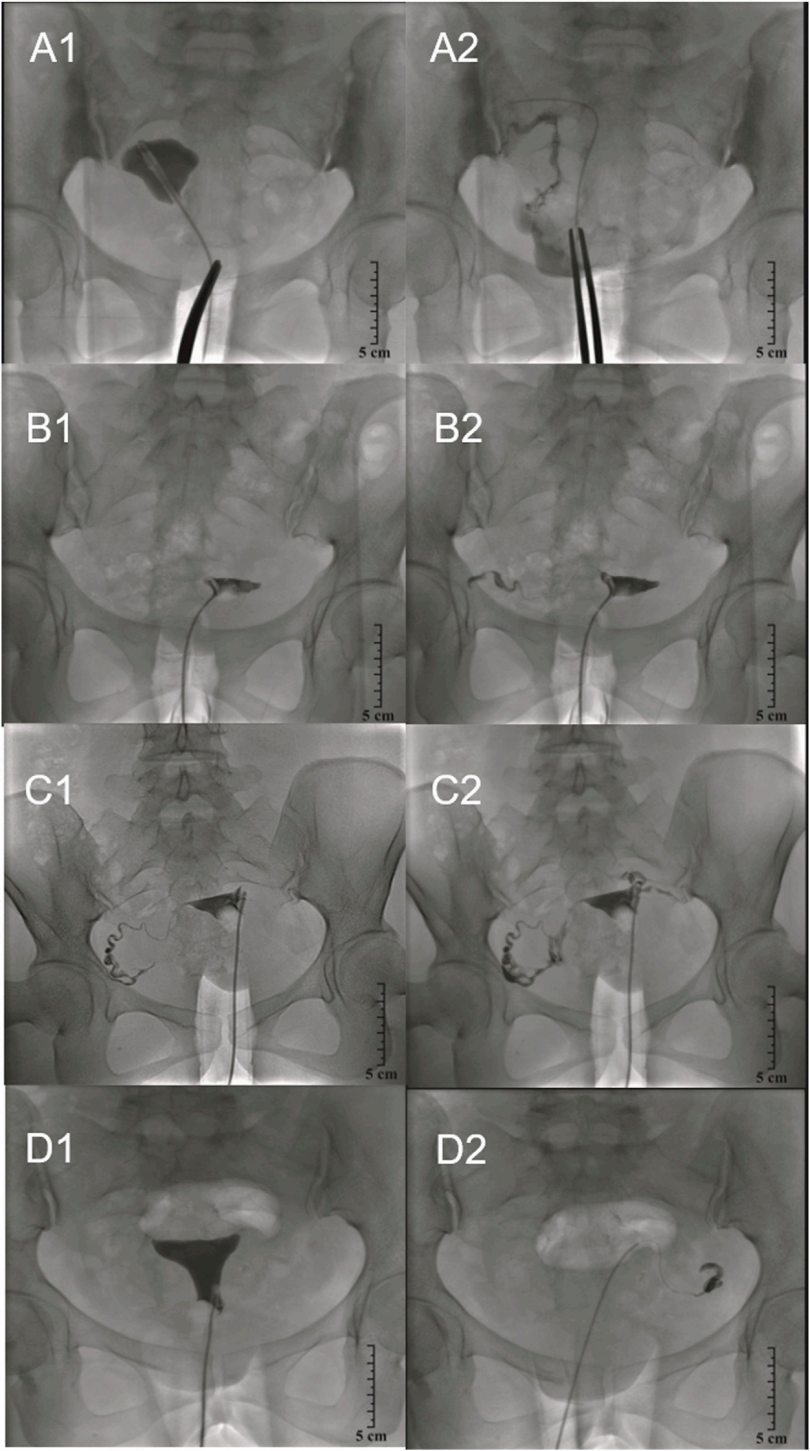
Pre- and post-injection salpingography. **(A)**. Control group. **(A1)**, The right fallopian tube completely obstructed at the preoperative stage. **(A2)**, The right fallopian tube partially unobstructed 1 day after interventional recanalization. **(B)**. Chitosan and danshen combined injection group (Group 2, 1:2 ratio) **(B1)**, The right fallopian tube completely obstructed at the pre-injection stage. **(B2)**, The right fallopian tube unobstructed 1 day after interventional recanalization. **(C)**. Chitosan and danshen combined injection group (Group C, 1:5 ratio) **(C1)**, The left fallopian tube completely obstructed at the preoperative stage. **(C2)**, The left fallopian tube unobstructed 1 day after interventional recanalization. **(D)**. Chitosan and danshen combined injection group (Group D, 1:10 ratio) **(D1)**, The left fallopian tube completely obstructed at the pre-injection stage. **D2**, The left fallopian tube unobstructed 1 day after interventional recanalization.

A total of 526 fallopian tubes were evaluated in 263 patients with salpingography preoperatively. 400 fallopian tubes were obstructed after follow-up. The tubal recanalization rates were highest in Group C (2 ml chitosan and 10 ml danshen, 97.1%), but the differences were not statistically significant (*p* = 0.17, *p* > 0.05, Table 5). Figure 3 shows a representative patient from each of the four treatment groups at the preoperative stage, 1 day after interventional recanalization.

**TABLE 5:** Analysis of fallopian tube recanalization rates 1 day after treatment.

	Preoperative obstructed fallopian tubes		Post-injection unobstructed fallopian tubes	Post-injection partially obstructed fallopian tubes	Post-injection obstructed fallopian tubes	Recanalization success rate (%)
Group A		102	92	4	6	90.2
Group B		97	93	3	1	95.9
Group C		102	99	3	0	97.1
Group D		99	93	4	2	93.9

Recanalization success rate = number of post-injection unobstructed tubes/number of preoperative obstructed tubes × 100%.

**FIGURE 4:**
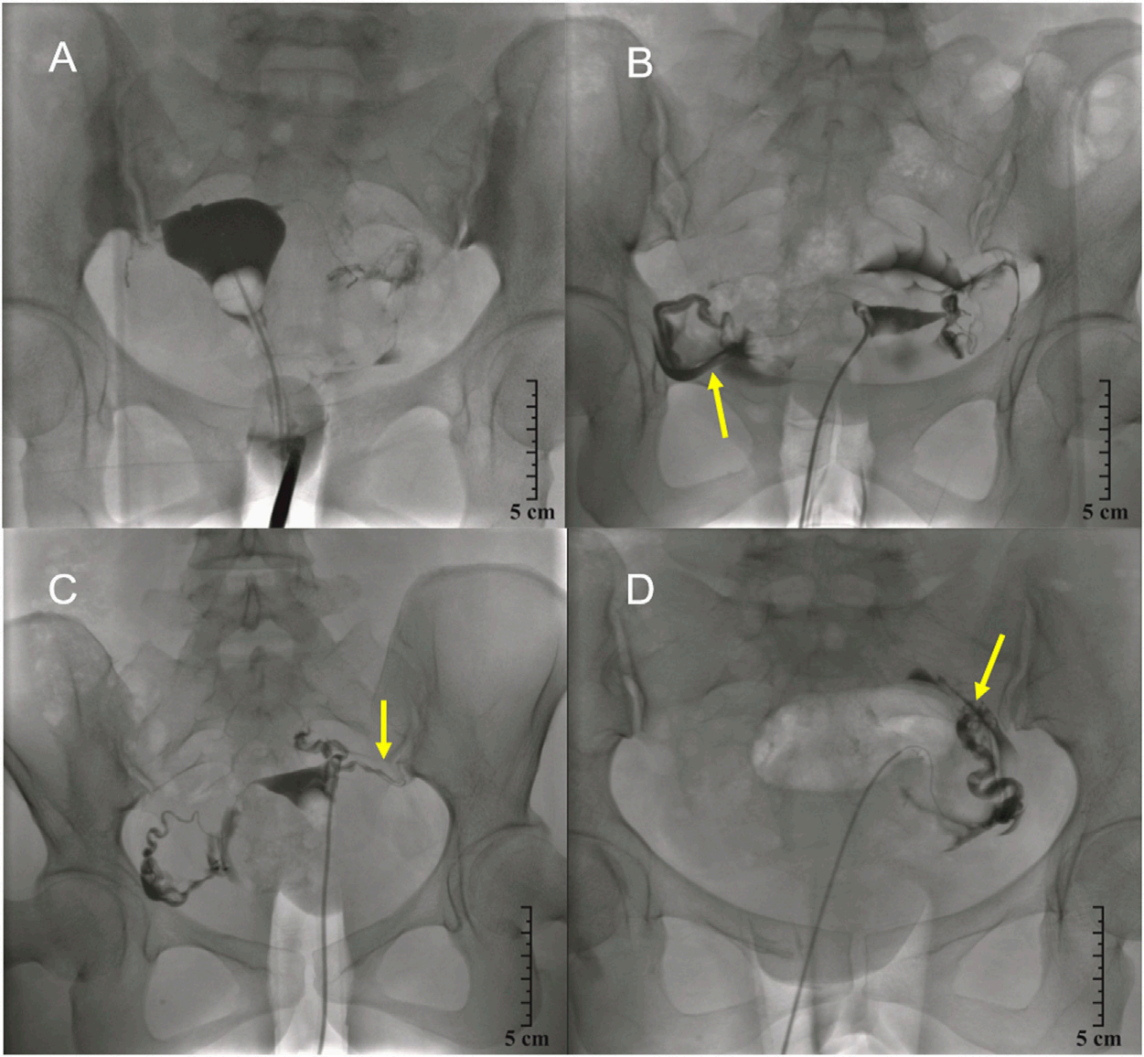
Selective salpingography showed fallopian tubes obstructed 1 year post-injection. **(A)**, Control group; **(B)**, Chitosan and Danshen combined injection group (1:2); **(C)**, Chitosan and Danshen injection group (1:5); and **(D)**, Chitosan and Danshen combined injection group (1:10). The yellow arrow shows the tube recanalized.

The tubal patency rate for patients receiving the combination of 2 ml chitosan and 10 ml danshen injection (Group C group, 1:5 mixture) at 1 year post-injection was substantially higher than that for Groups A, B, and D (94.2% vs. 38.6%, 73.5%, 68.5%), respectively; these differences were statistically significant (*p* = 0.0001, *p* < 0.05), as shown in Table 6. Figure 4 shows a representative patient from each of the four treatment groups 1 year after interventional recanalization.

**TABLE 6:** Analysis of tubal patency rates (in non-pregnant patients) 1-year post-injection (PI).

	Total fallopian tubes in non-pregnant patients	Unobstructed fallopian tubes PI	Partially obstructed fallopian tubes PI	Obstructed fallopian tubes PI	Recanalization success rate (%)
Group A	75	29	20	26	38.6
Group B	68	50	12	6	73.5
Group C	52	49	3	0	94.2
Group D	70	48	14	8	68.5

Patency rate = the number of unobstructed tubes/the number of patients at 1 year follow-up with patent fallopian tubes × 100%.

**FIGURE 5:**
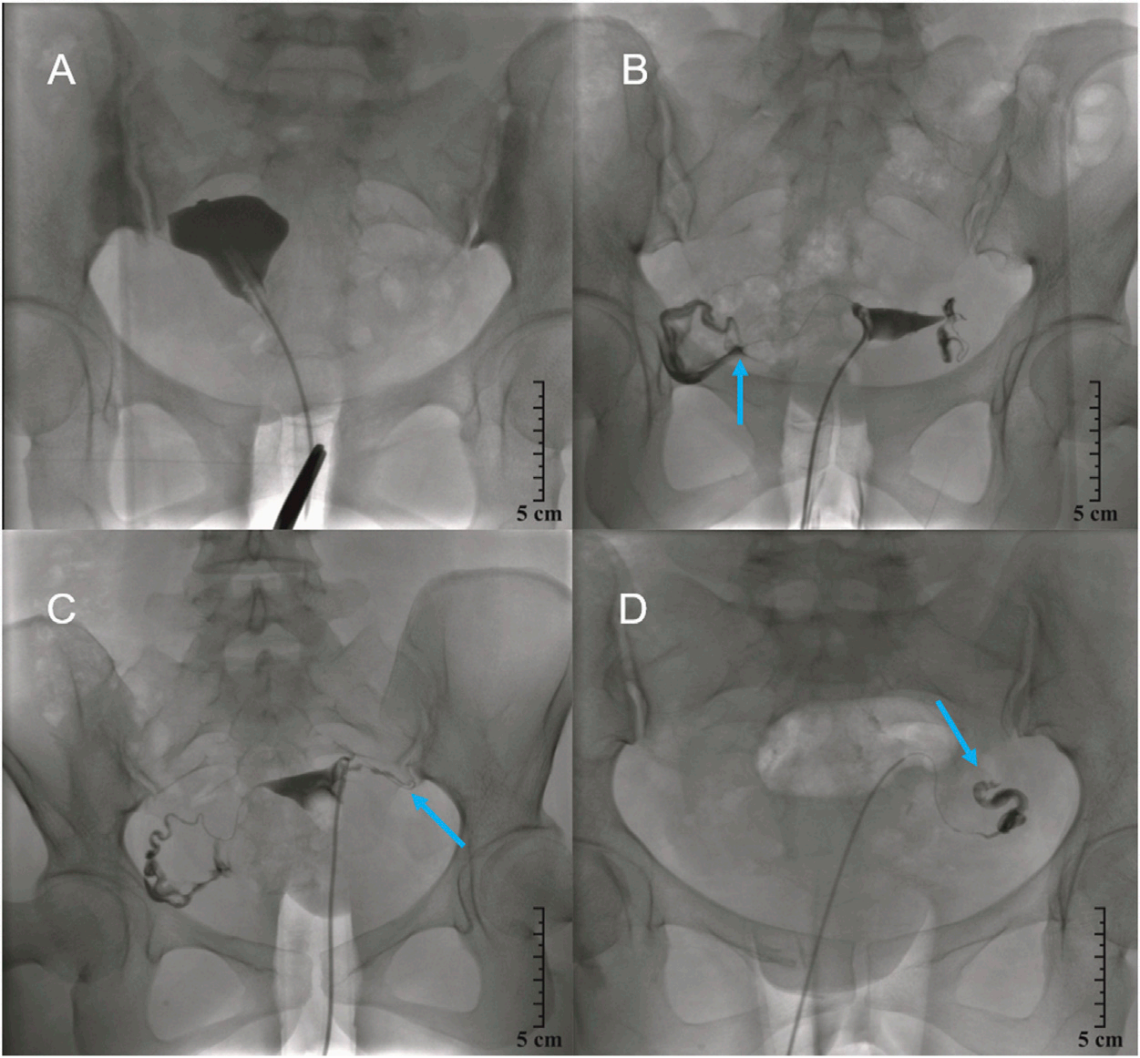
Selective salpingography showed fallopian tubes partially obstructed 3 years post-injection. **(A)**, Control group; **(B)**, Chitosan and Danshen combined injection group (1:2); **(C)**, Chitosan and Danshen combined injection group (1:5); and **(D)**, Chitosan and Danshen combined injection group (1:10). The blue arrow shows the tube partially recanalized.

The tubal patency rate in Group C (1:5 mixture of chitosan and danshen) at 3 years post-injection was also higher than that in Groups A, B, and D (87.3%vs. 31.5%, 64.1%, 50.7%), respectively; these differences were statistically significant (*p* = 0.0001, *p* < 0.05), as shown in Table 7. Figure 5 shows a representative patient from each of the four treatment groups 3 years after the interventional recanalization.

**TABLE 7:** Analysis of tubal patency rates (excluding pregnant patients) 3 years post-injection (PI).

	Total fallopian tubes in non-pregnant patients	Unobstructed fallopian tubes PI	Partially obstructed fallopian tubes PI	Obstructed fallopian tubes PI	Recanalization success rate (%)
Group A	73	23	21	29	31.5
Group B	74	47	19	8	64.1
Group C	78	67	9	2	87.3
Group D	69	35	22	12	50.7

Patency rate = the number of the post-injection unobstructed tubes/the number of follow-up patients the tubes × 100%.

#### 4.2.2 Pregnancy Rates at 1- and 3 years Post-Injection

The pregnancy rates at 1- and 3 years post-injection in Group C were 61.8% and 79.4%, respectively. These rates were higher than those of Group A (control group) (31.8% and 34.8%), as well as the other treatment groups. The differences were statistically significant (*p* = 0.0032 [year 1] and *p* = 0.0001 [year 3], *p* < 0.05), as shown in Table 8.

**TABLE 8:** Analysis of pregnancy rates in the patients 1- and 3 years post-injection (PI).

	Pregnancy 1 year PI	Pregnancy rate (%) 1 year PI	Pregnancy 3 years PI	Pregnancy rate (%) 3 years PI
Group A	21	31.8%	23	34.8%
Group B	26	40.1%	40	62.5%
Group C	42	61.8%	54	79.4%
Group D	25	38.5%	38	58.5%

Pregnancy rate = number of pregnant patients/follow-up number of patients × 100%.

## 5 Discussion

Chitosan is composed of arbitrarily distributed β-(1-4)-linked d-glucosamine (deacetylated unit) and N-acetyl-d-glucosamine (acetylated unit), which exhibits impressive characteristics, including mucoadhesive, biodegradable, and biocompatible properties. Chitosan has emerged as an essential element in the development of a nano-particulate delivery vehicle (Abolhasani et al., 2020; Babicheva et al., 2020; Palomino-Durand et al., 2020; Majcher et al., 2021; Yee Kuen and Masarudin, 2022).

Chitosan nanoparticles have loaded methylprednisolone sodium succinate to prolong the residence time and considerably inhibited the production of inflammatory cytokines, such as TNF-α and IL-6 (Ding et al., 2022). Great efficacy was found in the healing of diabetic skin wounds with alginate-and chitosan-based scaffolds (Cai and Li, 2020; Gaissler et al., 2021). Chitosan has also been shown to form a thermosensitive injectable hydrogel, and chitosan hydrogel-encapsulated mesenchymal stem cell exosomes show great potential in tissue repair (Wu et al., 2021). The redox-sensitive chitosan matrices can be used to realize synergistic photothermal-chemo therapy (Zhu et al., 2022). Celastrol-chitosan oligosaccharide (CSO) has also been found to be a promising delivery system for pancreatic cancer therapy (Zeng et al., 2022).

Tubal obstruction is one of the most common causes of female infertility, accounting for approximately 14%–45% of all female infertilities (Hanson et al., 2017; Shen et al., 2020). The recanalization rate of traditional tubal interventional procedures is approximately 90% (Knuttinen et al., 2014; Ikechebelu et al., 2018). But a high post-injection re-occlusion rate of 20–50% (Knuttinen et al., 2014) has been seen, as well as a low pregnancy rate of about 30% (Ikechebelu et al., 2018; Huang et al., 2019; Shen et al., 2020).

In this study, we developed a combined chitosan and danshen injection (China Invention Patent Number: ZL201510973669.3) Supplementary Figure S1. Chitosan, in correct proportions, can allow the release of drugs more slowly (Iftime et al., 2020). It can also inhibit inflammatory responses effectively (Liu et al., 2020), prevent post-injection adhesions (Liu et al., 2017) and fibrosis (Chung et al., 2016; Hassan et al., 2019), enhance collagen synthesis, promote revascularization (Shukla et al., 2021), and promote fallopian tube cilia repair. Cross-linked medium molecular weight chitosan nanoparticles are nontoxic, well-tolerated, and could, therefore, be a good candidate as a novel drug delivery system for improved therapeutic effects (Sethi et al., 2021). Especially, when the chitosan and danshen injection were combined, the effective constituent of chitosan and danshen injection was stable in our study.

We found an optimal 1:5 ratio of chitosan and danshen for injection that had a better efficacy to prevent tubal re-occlusion and improve the probability of a resultant pregnancy. We studied the stability of the 1:5 combined chitosan and danshen injection with high-performance liquid chromatography to determine the content changes of chitosan before and after mixing with danshen. We found that a ratio of 1:5 chitosan to danshen was more stable and produced better efficacy than the other ratios.

Compared with Group B (1:2 mixture), Group C with the 1:5 mixture was better at preventing post-injection fallopian tube re-adhesions. The analytic results might be due to the complex spatial structure of the chitosan macromolecule compound that can act as a carrier for the effective danshen compounds. With low doses of danshen, even if enough carrier is present, not enough danshen is available to provide a therapeutic effect on the fallopian tube.

Compared with Group D (1:10 mixture), Groups C and B were better at preventing post-injection fallopian tube re-adhesion with increasing doses of chitosan. The analytic results suggested that danshen itself reduces tissue adhesions; however, compared with Group C (1:5 mixture) and Group D (without enough chitosan to be effective as a carrier), danshen injections would be quickly metabolized. Danshen does not stay in the body for long periods, which would reduce its time in the fallopian tube and affect the overall prevention of fallopian tube re-adhesion. In addition, a large release of danshen in a short time could cause mucosal edema and hyperemia in fallopian tubes and slow down local microcirculation as seen in Group D, which is not conducive to the recovery of tubal function, with decreased patency rates seen 3 years after injection.

The tubal patency and pregnancy rates of Group C (1:5) were 87.3% and 79.4%, respectively, at 3 years post-injection and were significantly higher than those of the other groups. These results indicate that the combination of chitosan and danshen injection at a 1:5 ratio had positive effects on the probability of tubal patency and pregnancy after a long post-injection period.

Traditional Chinese medicine combined with interventional recanalization has a better effect on tubal obstruction than interventional recanalization alone, which may be caused by the regulation of inflammatory factors and the inhibition of fallopian tube inflammation (Liu et al., 2021). In addition, we found that the chitosan and danshen combined injection had a certain anti-inflammatory effect on the mucosa of fallopian tube in the pre-clinical study. Therapeutic Mechanism maybe promote the maturation of follicular cells by the TGF-β/SMAD and BMP/SMAD signaling pathways and increase the pregnancy rate (Liu et al., 2021). The new research (Marlow et al., 2021) showed the intrauterine pregnancy rate only was 31.4% with interventional recanalization alone and 59% using ozone treatment (He and Ma, 2015).The pregnancy rates of Group C (1:5) were 79.4% at 3 years post-injection in our study, which was higher than the other treatment. So combined chitosan and danshen injections observably increased pregnancy rates.

## 6 Conclusion

Combined chitosan and danshen injections are effective at preventing tubal re-obstruction and increasing pregnancy rates. The optimal concentration at 1:5 chitosan to danshen appears better than the other concentrations in treating and preventing fallopian tube obstruction.

## Data Availability

The original contributions presented in the study are included in the article/Supplementary Material, further inquiries can be directed to the corresponding authors.
