# Supplementary figures and images for: Dose-Effect Relationship of Chitosan and Danshen Combined Injection for Fallopian Tube Recanalization
Source: Front Pharmacol. 2022 Jun 14;13:935117. doi: 10.3389/fphar.2022.935117 (PMC9237208; doi:10.3389/fphar.2022.935117)

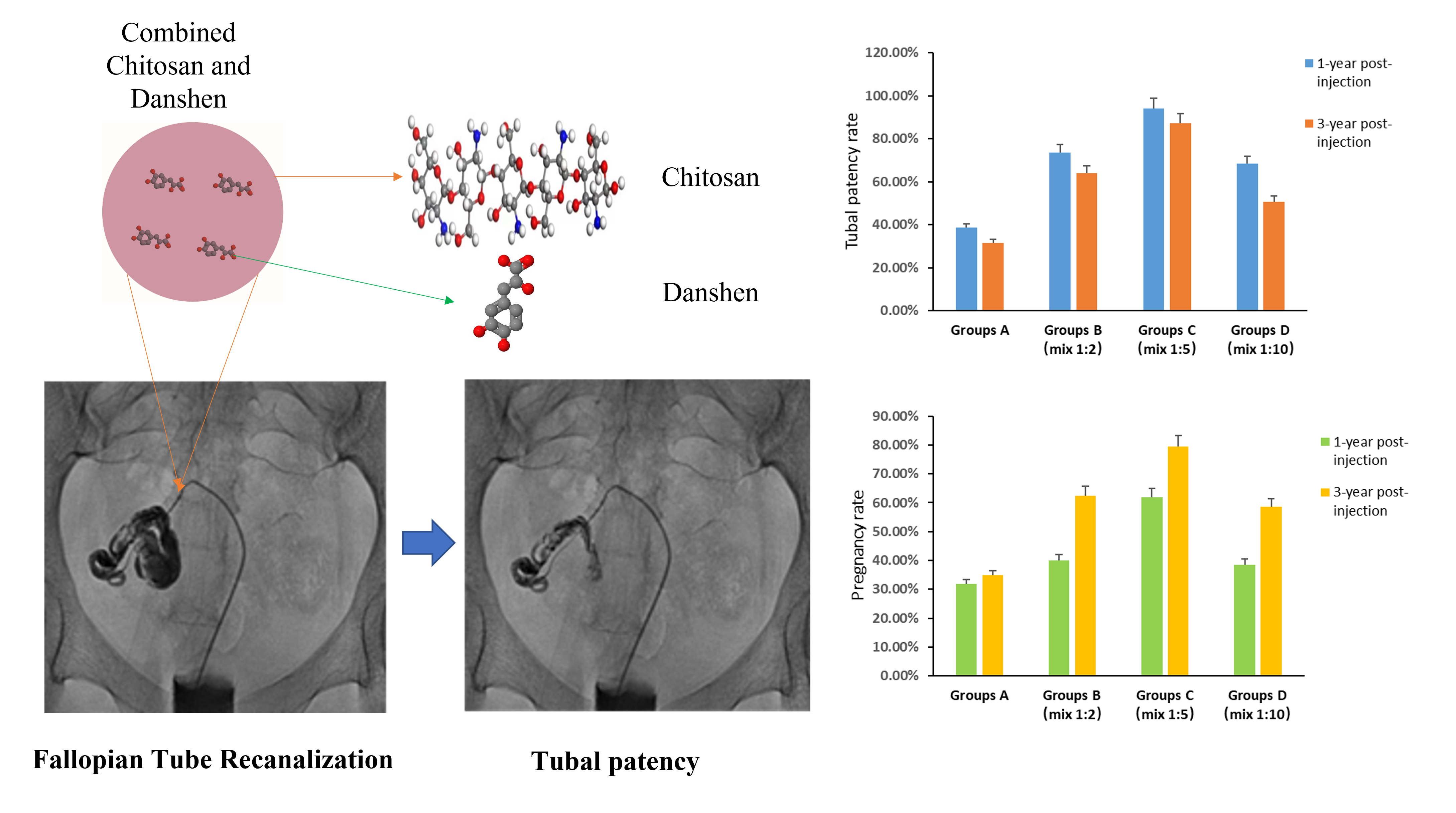

Supplement: Supplementary file 1 [file Image1.TIF]
